# Assessing the deviation from the inverse square law for orthovoltage beams with closed‐ended applicators

**DOI:** 10.1120/jacmp.v15i4.4893

**Published:** 2014-07-08

**Authors:** James Gräfe, Yannick Poirier, Ferenc Jacso, Rao Khan, Hong‐Wei Liu, J. Eduardo Villarreal‐Barajas

**Affiliations:** ^1^ Department of Medical Physics Tom Baker Cancer Centre Calgary AB; ^2^ Department of Physics and Astronomy University of Calgary Calgary AB; ^3^ CancerCare Manitoba Winnipeg MA; ^4^ Department of Oncology University of Alberta Edmonton AB; ^5^ Central Alberta Cancer Centre Red Deer AB Canada

**Keywords:** orthovoltage, extended source‐to‐surface distance, inverse square law, dose computation, kVDoseCalc

## Abstract

In this report, we quantify the divergence from the inverse square law (ISL) of the beam output as a function of distance (standoff) from closed‐ended applicators for a modern clinical orthovoltage unit. The divergence is clinically significant exceeding 3% at a 1.2 cm distance for 4 × 4 and $10\;\times \;10{\text{cm}}^{2}$ closed‐ended applicators. For all investigated cases, the measured dose falloff is more rapid than that predicted by the ISL and, therefore, causes a systematic underdose when using the ISL for dose calculations at extended SSD. The observed divergence from the ISL in closed‐ended applicators can be explained by the end‐plate scattering contribution not accounted for in the ISL calculation. The standoff measurements were also compared to the predictions from a home‐built kV dose computation algorithm, kVDoseCalc. The kVDoseCalc computation predicted a more rapid falloff with distance than observed experimentally. The computation and measurements agree to within 1.1% for standoff distances of 3 cm or less for $4\;\times \;4{\text{cm}}^{2}$ and $10\;\times \;10{\text{cm}}^{2}$ field sizes. The overall agreement is within 2.3% for all field sizes and standoff distances measured. No significant deviation from the ISL was observed for open‐ended applicators for standoff distances up to 10 cm.

PACS numbers: 87.55.‐x, 87.55.kh

## I. INTRODUCTION

Orthovoltage X‐ray tubes generally operate at 40‐350 kVp accelerating potentials. They are used to treat superficial skin cancers and bone disease, as well as benign tumors seated close to the skin. The characteristics of the percent depth‐dose (PDD) curves at these energies are such that the maximum dose ($d_{\text{max}}$) is at or near the surface. These PDDs present a more pronounced falloff with depth compared to megavoltage X‐ray beams. These characteristics allow orthovoltage beams to deliver high doses to superficial tumors, sparing underlying healthy tissues seated beyond the treatment target. The majority of orthovoltage treatments make use of X‐ray applicators (commonly called cones). These applicators are used to define the treatment field size by placing them directly on the patient surface, reducing the gap or standoff between the patient skin surface and the applicator. A lead (Pb) cutout can be used to define irregularly shaped fields and provide additional shielding. The standoff is defined as the distance between the patient skin surface and proximal end of the X‐ray applicator. In certain treatment situations, the standoff is unavoidable, such as in circumstances where patient curvature limits the applicator placement. In such circumstances, a standoff correction factor is used to account for the expected inverse square law (ISL) falloff of dose with distance from the applicator end.

Li et al.^(1)^ have reported divergence from the expected ISL for closed‐ended applicators. It is also recommended by the AAPM Task Group protocol for kilovoltage X‐ray beams (TG61) that this effect be taken in account.^(2)^ In this study, we have experimentally quantified the dose divergence from the inverse square law with standoff for a clinical Xstrahl‐300 orthovoltage unit and compare the results with computation using the kVDoseCalc calculation software^(3)^ in order to explain the sources of discrepancy. The standoff effect on monitor unit (MU) or timer calculations was also investigated.

## II. MATERIALS AND METHODS

### A. Clinical orthovoltage unit

The clinical unit investigated in this study was an Xstrahl‐300 X‐ray therapy system (Xstrahl Ltd., Camberley, UK). The Xstrahl‐300 unit can operate at potentials in the 40–300 kVp range. The Xstrahl‐300 unit was commissioned for clinical beams of 100, 150, and 200 kVp. Details of the clinical beams are shown in Table 1. The effective beam energy in Table 1 is defined as the mono‐energetic beam which produces the same half value layer (HVL). In addition to the added filtration shown in Table 1, all beams have an inherent filtration of 4 mm of Be. The X‐ray applicators are open‐ended and circular at 30 cm FSD (focus‐to‐skin distance). The open‐ended applicators have diameters of 1.5, 3, 4, 5, and 10 cm at the nominal 30 cm FSD. At 50 cm FSD, the applicators are closed‐ended and have square field size dimensions. The available closed‐ended applicators produce field sizes of 4×4, 6×6, 8×8, 10×10, 15×15, and $20\times 20\;{\text{cm}}^{2}$ at the nominal 50 cm FSD. The end plate of the closed‐ended applicators is composed of a 4 mm thick polymethyl methacrylate (PMMA) window (also known as acrylic).

**Table 1:** Nominal clinical beam parameters of the Xstrahl‐300 unit

*Potential (kVp)*	*HVL*	*Added Filtration*	*Effective Energy (keV)*	*mA*
100	3 mm Al	2 mm Al	35	26
150	6 mm Al	1 mm Al, 0.10 mm Cu	47	20
200	1 mm Cu	1 mm Al, 0.45 mm Cu	80	15

### B. In‐air measurements

The in‐air relative ionization measurements were performed with a Markus plane parallel ion chamber (PTW Frieburg, Germany; model N23343). The Markus chamber has a 0.055 cc nominal active volume and a 30 μm thick polyethylene entrance window. The 0.87 mm acrylic protective cap provided with the chamber is thick enough to remove any potential contaminating electrons; however, the addition of this extra material could act as an additional X‐ray scattering source. Therefore, this acrylic cap was not used. An acrylic ring was mounted on the chamber to allow a 1 mm offset between the applicator end plate and detector entrance window, avoiding any potential collisions between the applicator end plate and detector window. The electrode plate separation for the Markus detector is 2 mm. The effective point of measurement is the center of the detector air cavity^(2)^ (i.e., another 1 mm downstream of the applicator end plate). Therefore, the closest measurement position to the applicator end plate was 2 mm (due to electrode separation plus 1 mm offset). No physical offset was used for the open‐ended applicators, except for the fact that the effective point of measurement was at 1 mm from the applicator due to the detector plate separation. In regards to electron contamination, the continuous slowing down approximation (CSDA) range for electrons in water for 100, 150, and 200 keV electrons is approximately 140, 280, and 450 μm, respectively.^(4)^ These electrons have enough kinetic energy to penetrate the 30 μm thick polyethylene window of the Markus chamber. However these ranges correspond to the maximum X‐ray energy. At effective beam energies (listed in Table 1), the corresponding CSDA range for electrons are approximately 20, 40, and 98 μm, respectively. To determine if there was any significant electron contamination on our clinical unit, experiments were performed by placing a GAFCHROMIC EBT3 film (International Specialty Products, Wayne, NJ) as an electron absorber. EBT3 film has a manufacturer stated thickness of 250 μm and it is relatively tissue‐equivalent. The differences between the chamber response with and without the absorber, with the X‐ray attenuation taken into account, were used to estimate the electron contamination.

To determine if there is any dependence due to chamber size on the standoff assessment, measurements were also performed with a CC13 cylindrical ionization chamber (Scanditronix Wellhöfer, Nuremburg, Germany). The CC13 chamber has a 0.13 cc sensitive volume. This detector was chosen because of the similar volume compared to the 0.12 cc cylindrical ion chamber used by Li et al.^(1)^ The effective point of measurement for this chamber was taken as the center of the active volume and, therefore, the closest point of measurement for this chamber was 0.5 cm due to geometrical limitations of the detector size (limited by the detector radius and cable sheath). Since these are relative measurements performed in air, no effects due to chamber composition are expected since the beam quality in air does not change significantly within the short range of standoff distances used in the present study.

Ionizations in pC were recorded from a Max 4000 electrometer (Standard Imaging Inc., Middleton WI.). The ion chambers were positioned using a Standard Imaging 1D scanning arm. This scanning arm has a positional accuracy of ± 0.05 mm. Three measurements were performed at each position. The variation for these three measurements was found to be less than 0.3%. The ISL correction factor, or standoff correction (SOF) factor, is defined as:

(1) $$I_{ISL}={\left(\frac{FSD_{N}}{FSD_{N}+S}\right)}^{2}=SOF$$

where $FSD_{N}$ is the nominal FSD (30 cm for open‐ended and 50 cm for closed‐ended applicators), and *S* is the amount of standoff or distance from the $FSD_{N}$ in cm. The percentage difference between the calculated and measured relative ionization is defined as:

(2) $$Percentage\;difference=\left(\frac{I_{ISL}(S)-I_{meas}(S)}{I_{meas}(S)}\right)\times 100\%$$

where $I_{meas}(S)$ is the measured relative ionization normalized to zero standoff:

(3) $$I_{meas}(S)=\frac{I(S)}{I(0)}$$

The percentage differences measured represent the dosimetric error that occurs when only the ISL factor is used in the calculation of treatment monitor units.

### C. kVDoseCalc dose computations

Relative dose computations were performed using a validated^3^, ^5^, ^6^ hybrid kV dose calculation. kVDoseCalc computes the component of the dose deposited by primary photons using a deterministic model and the scatter component deposited by scattered photons using a stochastic biased Monte Carlo‐based technique.^(3)^ kV dose computations were performed to validate the experimental measurements for the closed‐ended applicators and provide a theoretical explanation for the observed phenomenon. The model of the beam geometry consisted of a flat beam where the fluence varied only due to divergence, and a field size defined by the nominal applicator size. The dose was computed similar to the measurement geometry; where air ($\rho =0.00129\;g/{\text{cm}}^{3}$) was used everywhere, except for the 4 mm acrylic end plate. The dose was computed to a small volume of air ($∼2\;{\text{mm}}^{3}$) as a function of standoff from the applicator end plate. The lower extremity of the end plate was placed at the nominal FSD; the atomic densities ($\text{atom}/{\text{cm}}^{3}$) were calculated using the nominal PMMA polymer composition $(C_{5}O_{2}H_{8})n$ and density ($1.18\;g/{\text{cm}}^{3}$). The spectrum of the 100 kVp beam was characterized by matching the measured (6.26 mm Al) HVL and nominal kVp with spectra generated by the third‐party freeware Spektr^(7)^ using the method described by Poirier et al.^(5)^ The highest kVp spectra that can be generated by Spektr is 140 kVp; for this reason the 150 and 200 kVp spectra were generated by inputting the nominal inherent 4 mm Be and added filtrations (provided in Table 1) into SpekCalc.^(8)^ The spectra generated by SpekCalc matched the measured HVL within 0.01 mm Al.

## III. RESULTS & DISCUSSION

### A. Open‐ended applicators

**Figure 1:**
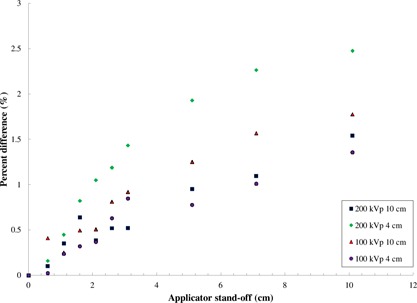
The percentage difference between the measured dose falloff and that expected from the ISL with distance from open‐ended circular applicators.

The percent difference as defined by Eq. (2) for the open‐ended applicators are shown in Fig. 1. Measurements were performed up to a standoff distance of 10 cm in order to demonstrate the overall trend. For standoff of up to 2 cm, the deviation between the measured relative dose and the expected value from ISL is ≤1% for the investigated beams. The largest percentage difference for the studied open‐ended applicators ≤1% is observed for the 4 cm diameter applicator at 200 kVp. It was found that the electron contamination was less than 0.7% for 200 kVp for the closed‐ended applicators and 1.1% for the open‐ended, and less than 0.2% for the 150 and 100 kVp beams for both open‐ended and closed‐ended applicators. The small, but systematic, underestimation of the standoff effect could be due to the electron contamination produced by this beam, which would be greatest near the applicator end and will fall off much faster than the ISL due to electron attenuation in air. However, this extreme difference is still small and has limited clinical significance, since it is unlikely that standoff for treatments with 4 cm applicators will exceed a few cm. Measurements were not performed for the smaller applicators of diameters 1.5 and 3 cm because it is highly unlikely that treatments will be performed with significant standoff for these field sizes. In clinical practice, these applicators are small enough to accommodate placement directly on the patient surface for most treatment sites. However, if there are treatment cases in which standoff is necessary due to patient comfort, the use of the ISL correction factor is acceptable for standoffs up to 10 cm.

### B. Closed‐ended applicators

**Figure 2:**
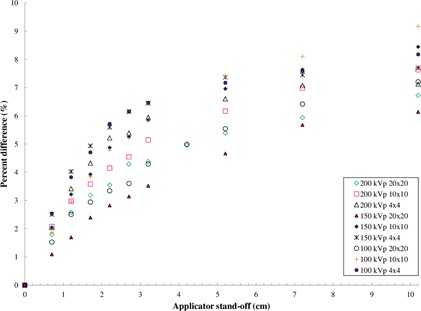
The percentage difference between the measured relative dose falloff and that expected from the ISL as a function of the distance from closed‐ended square applicators.

The deviation from the ISL law for the closed‐ended applicators is shown in Fig. 2. The Canadian Partnership for Quality Radiotherapy CPQR guideline for quality control on kilovoltage machines states the daily output tolerance should be 2%.^(9)^ Therefore, deviations of the machine output from the ISL as a function of standoff that exceeded this tolerance level were deemed clinically significant in this study. Furthermore, the deviations are systematic and can be accounted for with correction factors (see Results section E). For field sizes of 4×4 and $10\times 10\;{\text{cm}}^{2}$, the 3% deviation at a standoff of 1.2 cm is clinically significant. This represents the dosimetric error or underdosing that would occur if the ISL was the sole correction used in the dosimetry (MU) calculation. For a given kVp, the deviation is field size dependent for standoff distances of 5 cm and less, and the deviation increases with decreasing field size. For the 10×10 and $4\times 4\;{\text{cm}}^{2}$ field sizes, there is slight energy dependence; however, for the $20\times 20\;{\text{cm}}^{2}$ fields there is no apparent energy dependence observed. For the field size of $4\times 4\;{\text{cm}}^{2}$, the deviation is nearly 5% for a standoff of 2 cm for all investigated beam qualities.

### C. Dose computations

**Figure 3:**
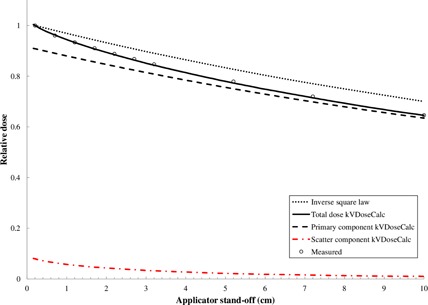
Comparison of the measured and computed relative total dose falloff as a function of applicator standoff for a 200 kVp beam with $10\times 10\;{\text{cm}}^{2}$ closed‐ended applicator. A much more rapid falloff is observed for the measured and computed data compared to the predicted falloff from the inverse square law. Also shown are the computed primary and scatter dose components.

A comparison of the measured and computed relative dose falloff in air is shown in Fig. 3 for a 200 kVp beam with $10\times 10\;{\text{cm}}^{2}$ close‐ended applicator. The measurements and computations were both normalized to the dose at 50.2 cm. This is the closest position achieved experimentally. The computation and experiment both demonstrate a more rapid dropoff of the dose with standoff compared to the inverse square law. The computation systematically predicts a slightly more rapid dose fall off compared to the measurements. For the data presented in Fig. 3, the agreement between experiment and computation varies between −0.8% and 0.2%. The computation and experimental measurements agree within 1.1% for standoff distances of 3 cm or less for $4\times 4\;{\text{cm}}^{2}$ and $10\times 10\;{\text{cm}}^{2}$ field sizes and over all energies. The overall agreement is within 2.3% for all field sizes and standoff distances measured in the Results section B.

The dose computed by kVDoseCalc can be separated into the primary and scattered components. The separate dose components are also shown in Fig. 3 for a 200 kVp beam with a $10\times 10\;{\text{cm}}^{2}$ closed‐ended applicator. The total dose to air falls off more rapidly than that predicted by the ISL. This can be explained by the X‐ray scattering in the 4 mm thick acrylic end plate. The end plate scattering component contributes approximately 10% to the total dose at the end plate, but drops off much more rapidly than the inverse square law. The primary dose component follows the ISL as expected; however, the addition of rapidly decreasing scatter component results in total dose which is lower than that expected by the ISL at the nominal 50 cm FSD.

**Figure 4:**
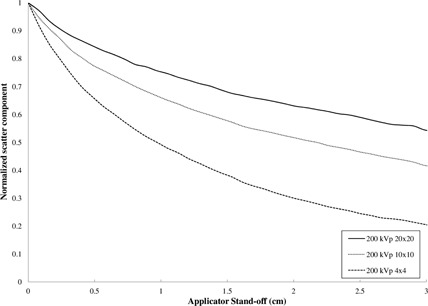
Normalized scatter component as a function of FS and applicator standoff for square closed‐ended applicators. The dropoff is much more pronounced with decreasing field size compared to the dropoff as a function of energy shown in Fig. 5.

The relative contribution of the scatter component to total dose at the end plate as a function of energy and field size (FS) is shown in Table 2. As expected, due to an increased scattering surface area of the end plate, the computation predicts a larger scatter contribution with increasing FS. Therefore, the falloff deviation decreases with increasing FS. The normalized scatter component falloff as a function of FS and standoff is shown in Fig. 4. The falloff becomes more pronounced with decreasing field size because of a reduction in the scattering area of the end plate with field size.

**Table 2:** Relative scatter contribution (%) to the total dose at the end plate as a function of field size and energy determined from kVDoseCalc

	*Accelerating Potential (kVp)*		
*Field size (cm^2^)*	*100*	*150*	*200*
$4\times 4\;{\text{cm}}^{2}$	10.9 %	10.2 %	8.6 %
$10\times 10\;{\text{cm}}^{2}$	12.5 %	11.6 %	10.3 %
$20\times 20\;{\text{cm}}^{2}$	13.4 %	12.3 %	10.8 %

**Figure 5:**
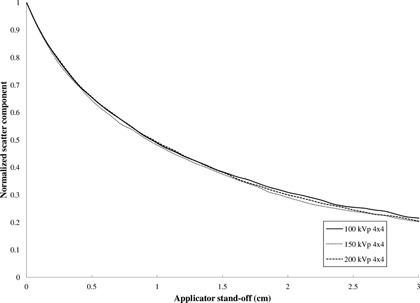
Normalized scatter component as a function of energy and applicator standoff for square closed‐ended applicators. The dropoff is much less pronounced as a function of energy compared to the FS dependence in Fig. 4.

The computation predicts a larger scatter component at the end plate with decreasing energy as shown in Table 2. This is as expected since the Compton scattering probability is inversely proportional to energy. Furthermore, Compton scattering is increasingly forward‐directed at higher energies, which means that fewer photons are scattered towards the central axis of the end plate.^(10)^ However, contrary to the above effect with field size, the normalized scatter component as a function of energy demonstrates the same proportional dropoff as shown in Fig. 5; therefore, only moderate energy dependence is observed, consistent with the experimental results.

### D. chamber comparison

The Markus chamber measurements were renormalized to 0.5 cm in order to compare with the CC13 measurements which cannot be measured at distances ≤0.5 cm due to the chamber radius. Over the measurement range of 0.5 cm to 10 cm, the percentage difference between the relative ionization for the CC13 and Markus chamber did not exceed 0.7%. The dropoff deviation from the ISL is, however, not as significant when the normalization is made at 0.5 cm. This is due to the fact that a significant contribution to the dose at the end plate (i.e., at 0 cm) is due to scattering in the end plate (see Results section C). Therefore, when normalizing at a point further downstream, the results will begin to follow more closely the ISL, as demonstrated by the computation results in Fig. 3 and discussed above. Therefore, we can conclude that there is no chamber‐related volume averaging or energy dependence for the measurement of the inverse square law divergence for the cylindrical and parallel plate ion chambers used in this study. However, the Markus chamber is a superior choice for these measurements since the effective point of measurement can be placed in close proximity with the endplate of the applicator.

The deviation observed for the closed‐ended applicators in Fig. 2 is larger than that observed by Li et al.^(1)^ This may be due to the thicker acrylic endplate for our applicators (4 mm compared to 3.2 mm^(1)^) and the use of the Markus chamber, which allows the effective point of measurement of the detector to be placed very close to the applicator end plate. In addition, Evans et al.^(11)^ observed no significant deviation from the ISL for closed‐ended applicators for a Gulmay D3300 unit. This, again, could be due to detector selection, since the effective point of measurement in their study was limited by the Farmer detector size to 4.3 mm. Furthermore, this effect is machine‐ and applicator composition‐specific, as mentioned by Li et al.^(1)^

### E. clinical implementation of the stand‐off correction

In order to implement the findings of our study into the clinical practice, it is important to consider the clinical impact of the standoff correction, as well as the possible errors that can occur due to its improper implementation. The standoff correction procedure should be efficient and made as simple as possible. The current procedure at our clinic is to calculate the number of monitor units required to deliver the daily tumor dose (DTD) using the following calculation:

(4) $$MU=\frac{DTD}{DWA\times SOF\times BSF}$$

where *DTD* is the prescribed daily tumor dose in cGy, *DWA* is the dose‐to‐water in‐air factor in cGy/MU, *SOF* is the standoff or standin correction factor (ISL correction), and *BSF* is the field size dependent backscatter factor.

**Figure 6:**
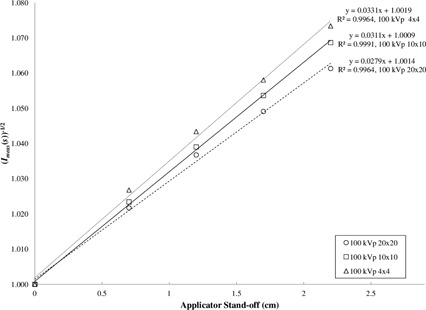
A plot of the square root of the inverse of the relative dose as function of standoff at 100 kVp for square closed‐ended applicators. The effective source position is determined from the inverse of the slope as defined in Khan.^(13)^

One method of correcting for the divergence of the inverse square law is to use the effective source position as proposed for electron calculations.^12^, ^13^ This is determined by plotting the square root of the inverse of the measured relative dose falloff as a function of standoff.^(13)^ This approach is shown for 100 kVp in Fig. 6. The inverse of the slope of these straight lines represents the effective source position. For use in clinical practice, the data were only fitted for standoff up to 2.2 cm.

The calculated effective source position as a function of energy and field size is shown in Table 3. The effective source position depends more on field size than the energy. Ideally a single value for the effective source position would be used. A value of 33 cm FSD provides reasonable agreement over all field sizes and energies, and is compared to the lookup table method below.

**Table 3:** The effective source position in cm as a function of energy (E) and field size (FS). The averages for either FS or E are also given. The dependence on field size is more significant than the dependence on E

$FS\;(cm^{2})$	*100 kVp*	*150 kVp*	*200 kVp*	*E: Average* ±SD
4×4	30.2	30.2	31.0	30.5±0.5
10×10	32.2	32.0	33.9	32.7±1.0
20×20	35.9	37.4	35.4	36.2±1.1
FS: Average ± SD	32.7±2.9	33.2±3.8	33.4±2.2	–

SD=standard deviation.

Another method to implement the standoff correction into clinical practice is to create a lookup table for the SOF as a function of FS. The lookup table is given in Table 4. The correction factor for the inverse square law alone is also given in the table. The standoff correction variation produced by the field size is small enough that linear interpolation for the other clinical field sizes of 6×6, 8×8, and $15\times 15\;{\text{cm}}^{2}$ could be performed safely. The agreement between the lookup table and using an effective source position of 33 cm is within ±1% for all field sizes investigated for distances up to 2 cm from the end plate; however, the lookup table provides the most accurate correction.

The measurements were performed at 0.2 cm from the end plate; however, in practice, the reduction in output is calculated from the nominal FSD of 50 cm with 0 cm standoff. The inverse square law correction of ${\left(50.2/50\right)}^{2}$ to normalize the measurements to a 0 cm standoff would introduce a 0.8% increase in the normalization point. However, according to kVdoseCalc, the correction factor varies from 1.7% to 2.8% for field sizes ranging from 20×20 to $4\times 4\;{\text{cm}}^{2}$, respectively. The small 0.8% correction was performed for the correction factors calculated in Table 4 to renormalize the data to zero standoff; however, based on computation we acknowledge that this may be an underestimation of up to 2%.

At extended SSD applications it is known that the percentage depth dose (PDD) increases with SSD. However the standoff distances of up to 5 cm typically encountered in the clinic will not change PDD by more than 1%–2%.^(1)^ The measured and computed divergence from the inverse square law is systematic and quantifiable. The statistical uncertainty in the dose computations was ≤1%. Based on measurement uncertainties (set up and reproducibility), we believe the standoff factors are accurate to within 1%, thus providing an accurate and simple correction method. The standoff correction shall be applied for closed‐ended applicators in order to reduce systematic dose errors for the orthovoltage X‐ray treatments. Our standoff findings reemphasize the results of Li et al.^(1)^ The standoff effect needs to be quantified for a given orthovoltage treatment unit; the radiation therapy planner can make an informed decision on the relevance of this parameter in their practice.

**Table 4:** Standoff correction factors as a function of field size for closed‐ended applicators. The data in this table are averages over all three energies. The standard deviation varied by less than 0.6% for the average over energy for each standoff position. The ISL correction factor at the nominal SSD of 50 cm is shown for comparison

	*Field Size (cm* ^*2*^ *)*			
*Standoff*	4×4	10×10	20×20	*ISL (50 cm)*
0	1.000	1.000	1.000	1.000
0.5	0.964	0.967	0.970	0.980
1.0	0.932	0.937	0.943	0.961
1.5	0.904	0.911	0.919	0.943
2.0	0.879	0.887	0.897	0.925
2.5	0.858	0.865	0.876	0.907
3.0	0.839	0.845	0.857	0.890
5.0	0.773	0.775	0.787	0.826

## IV. CONCLUSIONS

Based on our measurements and computations we recommend that the divergence from the inverse square law at extended SSD should be evaluated for all orthovoltage therapy units. We have determined field size dependent factors to account for the dose falloff as a function of distance from closed‐ended applicators for the Xstrahl 300 orthovoltage unit. We have also determined that a value of 33 cm as the effective source position for the nominal 50 cm FSD closed‐ended applicators provides an acceptable correction for all field sizes and energies within 2 cm of the applicator. Using only the inverse square law to account for a 1 cm gap from a closed‐ended applicator would result in a dosimetric error between 2%–3% for field sizes from 20×20 to $4\times 4\;{\text{cm}}^{2}$, respectively. At a 2 cm standoff, the dosimetric error would exceed 5% for a $4\times 4\;{\text{cm}}^{2}$ applicator. The deviations are systematic and are deemed clinically significant. The divergence from the inverse square law for closed‐ended applicators is a systematic effect and, if not taken into account, MU calculations will result in underdosing for orthovoltage treatments. Detectors, such as parallel plate ion chambers, that can measure the dose output in close proximity to the applicators are best suited to measure this effect. The open‐ended applicators follow the inverse square law to within 1% for standoff distances up to 2 cm and to within 2.5% for standoff distances up to 10 cm. Therefore, no additional corrections are necessary for open‐ended applicators.

## ACKNOWLEDGMENTS

We would like to thank Mauro Tambasco for creating and allowing us to use the kVDoseCalc kV dose computation software in this study. Thanks are extended to Joseph Madamesila for his assistance in performing some of the in‐air scanning measurements.

## Supporting information

Supplementary Material

Supplementary Material
