# Supplementary figures and images for: Assessing the deviation from the inverse square law for orthovoltage beams with closed‐ended applicators
Source: J Appl Clin Med Phys. 2014 Jul 8;15(4):356–66. doi: 10.1120/jacmp.v15i4.4893 (PMC5875524; doi:10.1120/jacmp.v15i4.4893)

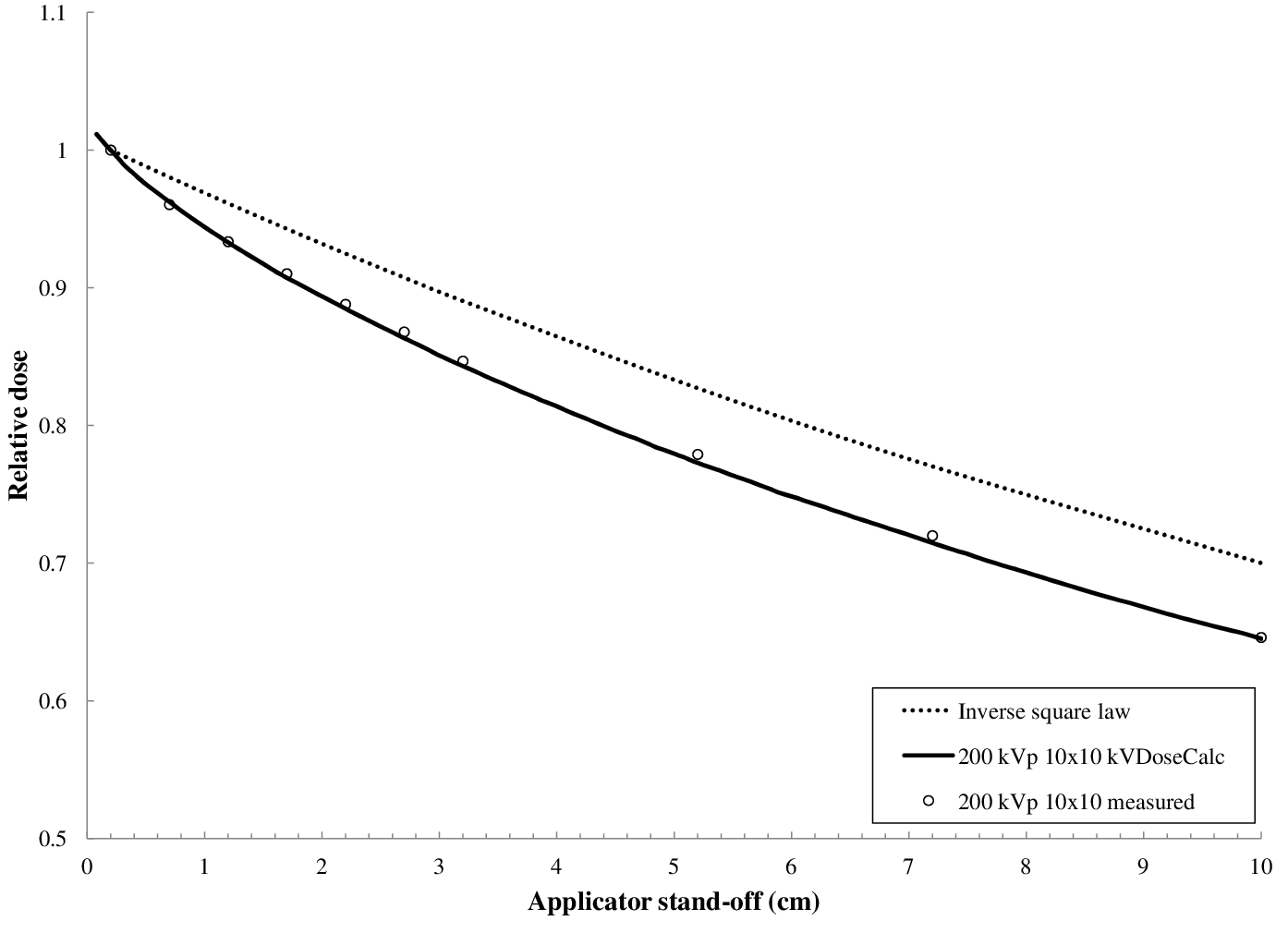

Supplement: Supplementary file 1 — Supplementary Material [file ACM2-15-356-s001.png]

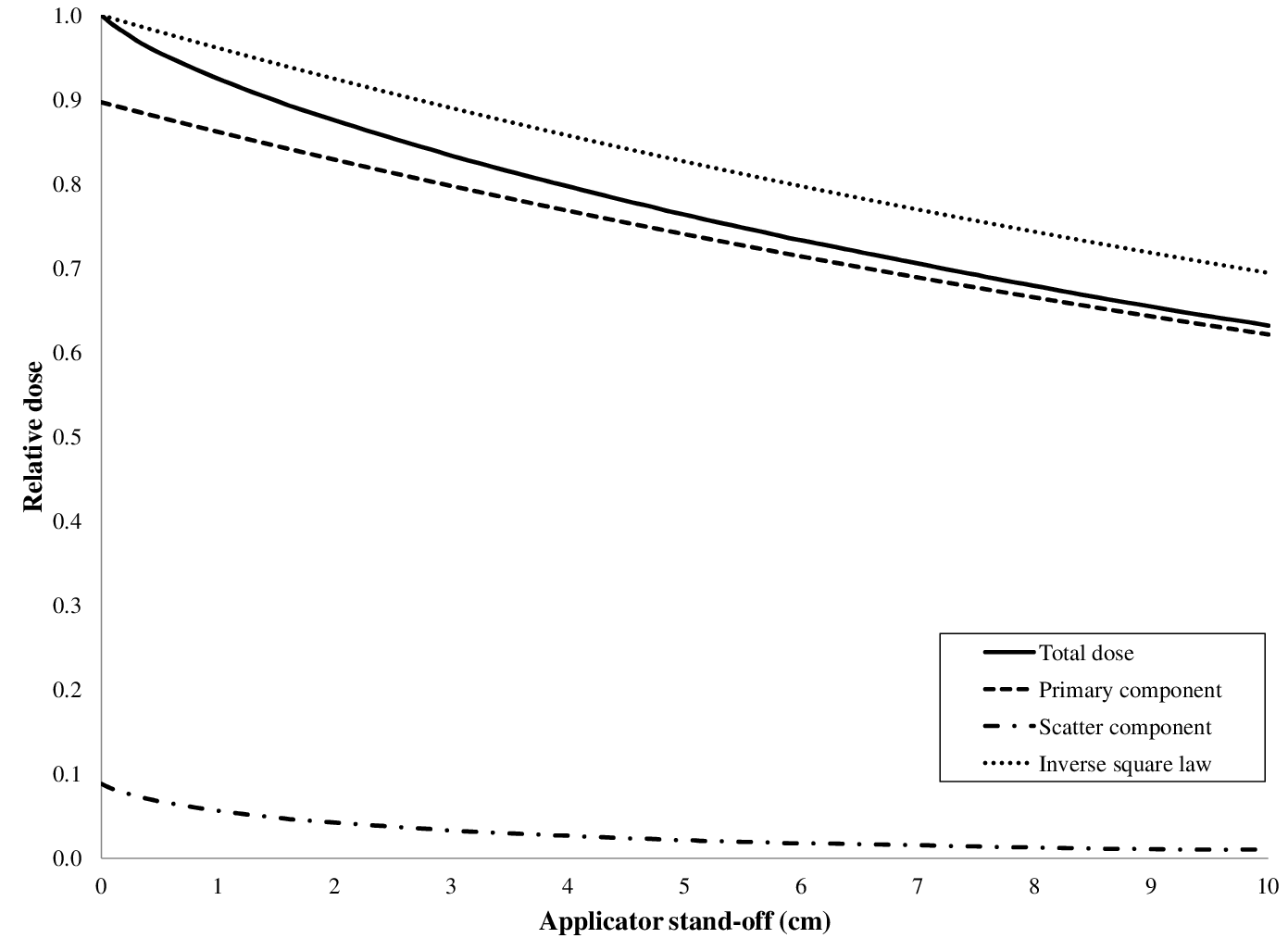

Supplement: Supplementary file 2 — Supplementary Material [file ACM2-15-356-s002.png]
